# 4-(4-Chloro­phen­yl)-1-methyl-3-trifluoro­methyl-1*H*-pyrazol-5-amine

**DOI:** 10.1107/S1600536810032435

**Published:** 2010-08-18

**Authors:** Xiao-Ping Jiang, Seik Weng Ng

**Affiliations:** a Department of Biology and Chemistry, Hunan University of Science and Engineering, Yongzhou, Hunan 425100, People’s Republic of China; b Department of Chemistry, University of Malaya, 50603 Kuala Lumpur, Malaysia

## Abstract

The five-membered ring of the title compound, C_11_H_9_ClF_3_N_3_, is almost planar (r.m.s. deviation = 0.002 Å) and the phenyl­ene ring is aligned at 44.8 (1)°. The N atom of the amino substituent shows a pyramidal geometry and is a hydrogen-bond donor to a Cl atom and to a ring N atom, which together generate a layer motif.

## Related literature

For the synthesis of the title compound, see: Coispeau (1977 ▶); Nishiwaki *et al.* (1995 ▶).
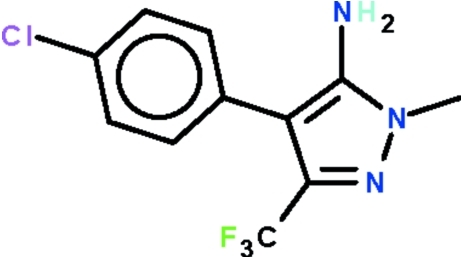


## Experimental

### 

#### Crystal data

C_11_H_9_ClF_3_N_3_
*M* *_r_* = 275.66
Monoclinic, 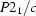

*a* = 5.8958 (5) Å
*b* = 16.8618 (13) Å
*c* = 12.1087 (10) Å
β = 98.459 (1)°
*V* = 1190.68 (17) Å^3^
*Z* = 4
Mo *K*α radiation
μ = 0.34 mm^−1^
*T* = 293 K
0.40 × 0.40 × 0.20 mm

#### Data collection

Bruker SMART area-detector diffractometer
Absorption correction: multi-scan (*SADABS*; Sheldrick, 1996 ▶) *T*_min_ = 0.864, *T*_max_ = 1.000
5716 measured reflections
2581 independent reflections
1769 reflections with *I* > 2σ(*I*)
*R*_int_ = 0.029

#### Refinement

*R*[*F* ^2^ > 2σ(*F* ^2^)] = 0.042
*wR*(*F* ^2^) = 0.119
*S* = 1.02
2581 reflections
172 parameters
2 restraints
H atoms treated by a mixture of independent and constrained refinement
Δρ_max_ = 0.32 e Å^−3^
Δρ_min_ = −0.26 e Å^−3^

### 

Data collection: *SMART* (Bruker, 1999 ▶); cell refinement: *SAINT* (Bruker, 1999 ▶); data reduction: *SAINT*; program(s) used to solve structure: *SHELXS97* (Sheldrick, 2008 ▶); program(s) used to refine structure: *SHELXL97* (Sheldrick, 2008 ▶); molecular graphics: *X-SEED* (Barbour, 2001 ▶); software used to prepare material for publication: *publCIF* (Westrip, 2010 ▶).

## Supplementary Material

Crystal structure: contains datablocks I, global. DOI: 10.1107/S1600536810032435/hg2700sup1.cif

Structure factors: contains datablocks I. DOI: 10.1107/S1600536810032435/hg2700Isup2.hkl

Additional supplementary materials:  crystallographic information; 3D view; checkCIF report

## Figures and Tables

**Table 1:** Hydrogen-bond geometry (Å, °)

*D*—H⋯*A*	*D*—H	H⋯*A*	*D*⋯*A*	*D*—H⋯*A*
N3—H1⋯Cl1^i^	0.88 (1)	2.65 (2)	3.413 (2)	146 (2)
N3—H2⋯N2^ii^	0.88 (1)	2.54 (2)	3.180 (3)	130 (2)

Symmetry codes: (i) 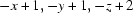; (ii) 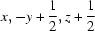.

**Fig. 1.**
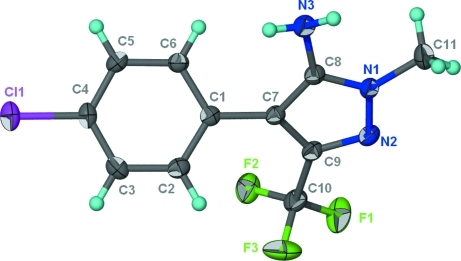
Thermal ellipsoid plot (Barbour, 2001) of C11H9ClF3N3 showing displacement ellipsoids at the 50% probability level. H-atoms are drawn as spheres of arbitrary radius.

## supplementary crystallographic information

### Comment

The title compound (Scheme I) is first mentioned in a synthesis by the cyclocondensation of hydrazines with 4,4,4-trifluoro-2-arylacetoacetonitriles in the context of colorants for polyacrylonitriles (Coispeau, 1977). The structure has been eluciated by carbon-13 NMR spectroscopy (Nishiwaki *et al.*, 1995). We have used a modification of the published procedure to synthesize the compound, which is intended for further study on its pharmaceutical activity. There are no other reports on this compound aside from these studies.

### Experimental

Sodium metal (0.35 g, 15 mmol) was dissolved in absolute ethanol (50 ml). To this solution was added 2-(4-chlorophenyl)acetonitrile (1.52 g, 10 mmol) followed by ethyl trifluoroacetate (1.42 g, 10 mmol). The solution was heated for 3 h. The solution was concentrated under vacuum. To the residue was added acetic acid (20 ml) followed by methylhydrazine (0.55 g, 12 mmol). The mixture was stirred for 12 h. The solution was again concentrated under vacuum. The residue was treated with water (30 ml) and the organic compound was extracted with ethyl acetate. The organic phase was washed with saturated sodium bicarbonate (230 ml) and then dried over sodium sulfate. The solvent was removed and the residue was chromatographed on a silica gel column with ethyl acetate:petroleum ether (1:10) as eluant. This gave 2 g (70%) of product as a yellow solid, which was recrystallized from ethyl acetate.

### Refinement

Carbon bound H-atoms were positioned geometrically and refined using the riding model, and with C–H = 0.93 to 0.96 Å and *U*(H) set to 1.2–1.5 *U*_eq_(C). The amino H-atoms were located in a difference Fourier map, and were refined with a distance restraint of N–H 0.88±0.01 Å; their temperature factors were refined.

### Crystal data

**Table:**

C_11_H_9_ClF_3_N_3_	*F*(000) = 560
*M**_r_* = 275.66	*D*_x_ = 1.538 Mg m^−^^3^
Monoclinic, *P*2_1_/*c*	Mo *K*α radiation, λ = 0.71073 Å
Hall symbol: -P 2ybc	Cell parameters from 2152 reflections
*a* = 5.8958 (5) Å	θ = 2.5–26.6°
*b* = 16.8618 (13) Å	µ = 0.34 mm^−^^1^
*c* = 12.1087 (10) Å	*T* = 293 K
β = 98.459 (1)°	Block, yellow
*V* = 1190.68 (17) Å^3^	0.40 × 0.40 × 0.20 mm
*Z* = 4	

### Data collection

**Table:**

Bruker SMART area-detector diffractometer	2581 independent reflections
Radiation source: fine-focus sealed tube	1769 reflections with *I* > 2σ(*I*)
graphite	*R*_int_ = 0.029
φ and ω scans	θ_max_ = 27.0°, θ_min_ = 2.1°
Absorption correction: multi-scan (*SADABS*; Sheldrick, 1996)	*h* = −7→7
*T*_min_ = 0.864, *T*_max_ = 1.000	*k* = −21→10
5716 measured reflections	*l* = −15→12

### Refinement

**Table:**

Refinement on *F*^2^	Primary atom site location: structure-invariant direct methods
Least-squares matrix: full	Secondary atom site location: difference Fourier map
*R*[*F*^2^ > 2σ(*F*^2^)] = 0.042	Hydrogen site location: inferred from neighbouring sites
*wR*(*F*^2^) = 0.119	H atoms treated by a mixture of independent and constrained refinement
*S* = 1.02	*w* = 1/[σ^2^(*F*_o_^2^) + (0.0629*P*)^2^ + 0.1525*P*] where *P* = (*F*_o_^2^ + 2*F*_c_^2^)/3
2581 reflections	(Δ/σ)_max_ = 0.001
172 parameters	Δρ_max_ = 0.32 e Å^−^^3^
2 restraints	Δρ_min_ = −0.26 e Å^−^^3^

### Fractional atomic coordinates and isotropic or equivalent isotropic displacement parameters (Å^2^)

**Table:**

	*x*	*y*	*z*	*U*_iso_*/*U*_eq_	
Cl1	0.24293 (10)	0.63048 (3)	0.86611 (5)	0.03743 (19)	
F1	0.9033 (3)	0.36727 (9)	0.43732 (12)	0.0619 (5)	
F2	0.7674 (3)	0.46344 (8)	0.52371 (12)	0.0503 (4)	
F3	0.5700 (3)	0.35880 (11)	0.48636 (14)	0.0685 (5)	
N1	1.1139 (3)	0.27925 (10)	0.73828 (14)	0.0272 (4)	
N2	1.0499 (3)	0.29559 (10)	0.62838 (14)	0.0292 (4)	
N3	1.0445 (3)	0.31883 (12)	0.91929 (16)	0.0314 (4)	
H1	0.929 (3)	0.3386 (15)	0.948 (2)	0.054 (8)*	
H2	1.087 (4)	0.2714 (8)	0.944 (2)	0.044 (7)*	
C1	0.6890 (4)	0.43079 (11)	0.77159 (17)	0.0238 (5)	
C2	0.4645 (4)	0.43995 (12)	0.71925 (18)	0.0283 (5)	
H2A	0.4060	0.4049	0.6629	0.034*	
C3	0.3258 (4)	0.50032 (13)	0.74945 (19)	0.0293 (5)	
H3	0.1767	0.5063	0.7129	0.035*	
C4	0.4114 (4)	0.55085 (12)	0.83380 (19)	0.0271 (5)	
C5	0.6307 (4)	0.54242 (12)	0.89124 (18)	0.0282 (5)	
H5	0.6846	0.5763	0.9499	0.034*	
C6	0.7681 (4)	0.48252 (12)	0.85957 (18)	0.0266 (5)	
H6	0.9161	0.4764	0.8975	0.032*	
C7	0.8443 (4)	0.37063 (11)	0.73603 (18)	0.0239 (4)	
C8	0.9973 (3)	0.32257 (12)	0.80477 (18)	0.0253 (5)	
C9	0.8878 (4)	0.35046 (12)	0.62864 (18)	0.0267 (5)	
C10	0.7831 (4)	0.38467 (14)	0.5202 (2)	0.0377 (6)	
C11	1.2992 (4)	0.22382 (14)	0.7729 (2)	0.0398 (6)	
H11A	1.4002	0.2456	0.8349	0.060*	
H11B	1.3831	0.2146	0.7119	0.060*	
H11C	1.2369	0.1746	0.7948	0.060*	

### Atomic displacement parameters (Å^2^)

**Table:**

	*U*^11^	*U*^22^	*U*^33^	*U*^12^	*U*^13^	*U*^23^
Cl1	0.0397 (3)	0.0332 (3)	0.0428 (4)	0.0094 (2)	0.0172 (3)	0.0028 (2)
F1	0.0999 (14)	0.0632 (11)	0.0261 (8)	0.0360 (9)	0.0205 (8)	0.0060 (7)
F2	0.0765 (11)	0.0380 (8)	0.0359 (9)	0.0191 (7)	0.0067 (7)	0.0071 (6)
F3	0.0630 (11)	0.0885 (13)	0.0445 (10)	−0.0080 (9)	−0.0238 (9)	−0.0024 (9)
N1	0.0322 (10)	0.0243 (9)	0.0245 (10)	0.0041 (8)	0.0025 (8)	−0.0016 (7)
N2	0.0370 (11)	0.0260 (9)	0.0242 (10)	−0.0007 (8)	0.0037 (8)	−0.0031 (7)
N3	0.0414 (12)	0.0300 (11)	0.0223 (10)	0.0046 (9)	0.0023 (9)	0.0029 (8)
C1	0.0276 (11)	0.0209 (10)	0.0230 (11)	−0.0014 (8)	0.0040 (9)	0.0029 (8)
C2	0.0305 (12)	0.0269 (11)	0.0273 (12)	−0.0045 (9)	0.0036 (9)	−0.0009 (9)
C3	0.0244 (11)	0.0300 (11)	0.0334 (13)	−0.0007 (9)	0.0039 (9)	0.0074 (9)
C4	0.0304 (12)	0.0221 (10)	0.0316 (12)	0.0037 (9)	0.0139 (10)	0.0055 (9)
C5	0.0333 (12)	0.0274 (11)	0.0243 (11)	−0.0033 (9)	0.0057 (9)	−0.0041 (9)
C6	0.0272 (11)	0.0258 (11)	0.0259 (12)	−0.0005 (9)	0.0007 (9)	−0.0002 (9)
C7	0.0272 (11)	0.0212 (10)	0.0232 (11)	−0.0017 (8)	0.0030 (9)	−0.0003 (8)
C8	0.0283 (11)	0.0205 (10)	0.0271 (12)	−0.0035 (8)	0.0034 (9)	−0.0004 (9)
C9	0.0312 (11)	0.0228 (10)	0.0257 (12)	−0.0028 (9)	0.0026 (9)	−0.0028 (9)
C10	0.0496 (15)	0.0362 (13)	0.0260 (13)	0.0060 (11)	0.0014 (11)	−0.0025 (10)
C11	0.0408 (14)	0.0353 (13)	0.0425 (15)	0.0127 (11)	0.0038 (11)	−0.0030 (11)

### Geometric parameters (Å, °)

**Table:**

Cl1—C4	1.749 (2)	C2—C3	1.388 (3)
F1—C10	1.344 (3)	C2—H2A	0.9300
F2—C10	1.333 (3)	C3—C4	1.368 (3)
F3—C10	1.336 (3)	C3—H3	0.9300
N1—C8	1.348 (3)	C4—C5	1.382 (3)
N1—N2	1.357 (2)	C5—C6	1.384 (3)
N1—C11	1.452 (3)	C5—H5	0.9300
N2—C9	1.331 (3)	C6—H6	0.9300
N3—C8	1.375 (3)	C7—C8	1.394 (3)
N3—H1	0.88 (1)	C7—C9	1.404 (3)
N3—H2	0.88 (1)	C9—C10	1.483 (3)
C1—C2	1.389 (3)	C11—H11A	0.9600
C1—C6	1.402 (3)	C11—H11B	0.9600
C1—C7	1.473 (3)	C11—H11C	0.9600
			
C8—N1—N2	112.53 (17)	C5—C6—H6	119.3
C8—N1—C11	127.18 (19)	C1—C6—H6	119.3
N2—N1—C11	120.18 (18)	C8—C7—C9	102.82 (18)
C9—N2—N1	103.59 (17)	C8—C7—C1	126.98 (19)
C8—N3—H1	109.5 (19)	C9—C7—C1	130.08 (19)
C8—N3—H2	113.0 (17)	N1—C8—N3	122.16 (19)
H1—N3—H2	114 (2)	N1—C8—C7	107.49 (19)
C2—C1—C6	117.73 (19)	N3—C8—C7	130.2 (2)
C2—C1—C7	122.32 (18)	N2—C9—C7	113.57 (19)
C6—C1—C7	119.94 (19)	N2—C9—C10	118.3 (2)
C3—C2—C1	121.3 (2)	C7—C9—C10	128.1 (2)
C3—C2—H2A	119.3	F2—C10—F3	105.6 (2)
C1—C2—H2A	119.3	F2—C10—F1	106.7 (2)
C4—C3—C2	119.1 (2)	F3—C10—F1	105.95 (19)
C4—C3—H3	120.5	F2—C10—C9	112.43 (19)
C2—C3—H3	120.5	F3—C10—C9	113.2 (2)
C3—C4—C5	121.79 (19)	F1—C10—C9	112.3 (2)
C3—C4—Cl1	119.06 (17)	N1—C11—H11A	109.5
C5—C4—Cl1	119.10 (17)	N1—C11—H11B	109.5
C4—C5—C6	118.5 (2)	H11A—C11—H11B	109.5
C4—C5—H5	120.7	N1—C11—H11C	109.5
C6—C5—H5	120.7	H11A—C11—H11C	109.5
C5—C6—C1	121.4 (2)	H11B—C11—H11C	109.5
			
C8—N1—N2—C9	−0.4 (2)	N2—N1—C8—C7	0.5 (2)
C11—N1—N2—C9	−176.89 (19)	C11—N1—C8—C7	176.7 (2)
C6—C1—C2—C3	2.8 (3)	C9—C7—C8—N1	−0.3 (2)
C7—C1—C2—C3	−175.81 (19)	C1—C7—C8—N1	−176.80 (19)
C1—C2—C3—C4	−1.1 (3)	C9—C7—C8—N3	175.8 (2)
C2—C3—C4—C5	−1.3 (3)	C1—C7—C8—N3	−0.6 (4)
C2—C3—C4—Cl1	176.15 (16)	N1—N2—C9—C7	0.2 (2)
C3—C4—C5—C6	2.0 (3)	N1—N2—C9—C10	177.86 (18)
Cl1—C4—C5—C6	−175.48 (16)	C8—C7—C9—N2	0.1 (2)
C4—C5—C6—C1	−0.2 (3)	C1—C7—C9—N2	176.4 (2)
C2—C1—C6—C5	−2.1 (3)	C8—C7—C9—C10	−177.3 (2)
C7—C1—C6—C5	176.54 (19)	C1—C7—C9—C10	−1.0 (4)
C2—C1—C7—C8	−138.4 (2)	N2—C9—C10—F2	−134.0 (2)
C6—C1—C7—C8	43.0 (3)	C7—C9—C10—F2	43.3 (3)
C2—C1—C7—C9	46.1 (3)	N2—C9—C10—F3	106.4 (2)
C6—C1—C7—C9	−132.4 (2)	C7—C9—C10—F3	−76.3 (3)
N2—N1—C8—N3	−176.08 (18)	N2—C9—C10—F1	−13.6 (3)
C11—N1—C8—N3	0.1 (3)	C7—C9—C10—F1	163.7 (2)

### Hydrogen-bond geometry (Å, °)

**Table:**

*D*—H···*A*	*D*—H	H···*A*	*D*···*A*	*D*—H···*A*
N3—H1···Cl1^i^	0.88 (1)	2.65 (2)	3.413 (2)	146 (2)
N3—H2···N2^ii^	0.88 (1)	2.54 (2)	3.180 (3)	130 (2)

Symmetry codes: (i) −*x*+1, −*y*+1, −*z*+2; (ii) *x*, −*y*+1/2, *z*+1/2.
